# Expression of E- Cadherin and Levels of Dysplasia in Oral Leukoplakia - A Prospective Cohort Study

**DOI:** 10.31557/APJCP.2020.21.2.405

**Published:** 2020

**Authors:** I Ilangani Sathish, Kannan Asokan, Krithika C L, Arvind Ramanathan

**Affiliations:** 1 *Department of Oral Medicine and Radiology, SRM Dental College, Ramapuram, Chennai, Tamil Nadu, *; 2 *Enable Biolabs, Madurai Meenakshipuram Extension, Urapakkam, Chennai, India. *

**Keywords:** E – cadherin, oral leucoplakia, cancer progression, PCR

## Abstract

**Aim:**

Enormous attempts have been made to develop and establish markers that determines the susceptibility of potentially malignant tissues to transform to oral cancer. E - cadherin encoded by CDH1 gene is a protein which plays an important role in cellular adhesion. This study aimed to assess the relationship between the expression of E- cadherin and different grades of epithelial dysplasia in oral leukoplakia.

**Materials and Methods:**

Tumour biopsies from fifty leukoplakia patients was collected. Half of the tissue was sent for histopathological examination and other half was subjected to see E - cadherin expression by real time PCR.

**Results:**

On assessing, the expression of E - cadherin was found to be high in samples with mild dysplasia followed by samples with moderate dysplasia. Samples with severe dysplastic feature showed least expression of E - cadherin. All statistical analyses were performed using Statistical Package for Social science (SPSS) and was proven that there is significant decrease in the expression of E - cadherin as the degree of dysplasia increases with a p value 0.001 and confidence interval 95%.

**Conclusion:**

We conclude that loss of E - cadherin can be used as a tumour marker that could determine the susceptibility of normal and potentially malignant tissues to transform into oral cancers. To generalise our results, further prospective studies with a large sample size using quantitative real time PCR to read the gene expression should be carried out at multi centre levels.

## Introduction

The World Health Organization (WHO) defines premalignant lesions as, “a morphologically altered tissue in which oral cancer is more likely to occur than its apparently normal counterpart ” and premalignant condition as, “a generalized state associated with significantly increased risk of cancer”. Oral premalignant lesions include Leukoplakia, Erythroplakia and palatal lesions of reverse smokers and oral premalignant conditions include Oral sub mucous fibrosis, Lichen planus, Epidermolysis bullosa, Discoid lupous erythematosis, etc,. Collectively Oral potentially malignant disorders (OPMD) are defined as, “risk of malignancy being present in a lesion or condition either at the time of initial diagnosis or at a future date”. 

Increased attention has been focused on leukoplakia due to high incidence and its potential for malignant transformation. Malignant transformation rates depends on part of the population, gender, tobacco usage and grading of dysplasia. Oral epithelial dysplastic changes plays an important role in progression from normal to malignant lesions. These dysplastic changes are considered to be precursors for malignant changes. WHO grades oral epithelial dysplasia as mild, moderate and severe depending on cellular atypia and thickness of dysplastic layers compared to total epithelial height. Therefore, assessing the accurate degree of dysplasia reflecting malignant disease is challenging and creates barriers in prediction and management of such lesions. Histopathological grading of epithelial dysplasia remains one of the most clinically important predictors of the malignant potential of a lesion.

There have been numerous attempts to develop tumour markers to determine the susceptibility of transformation of normal cells to malignant cells. Malignant transformation is associated with decreased differentiation and loss in epithelial phenotype. During this process, epithelial to mesenchymal transition (EMT) occurs in which epithelial cells reorganize their cytoskeleton acquiring a mesenchymal phenotype. Expression of mesenchymal genes is accompanied by loss of epithelial characteristics including loss of epithelial cell polarity, decreased cellular adhesion and increased mobility. Cell adhesion plays an important structural role with stratified squamous epithelium. There is a strong relationship between reduced expression of adhesion molecules and decreased differentiation and invasiveness of the lesion. 

E-cadherin (E-cad) is a tumour suppressor gene that is expressed in epithelial tissues. It is a cell membrane associated protein involved in cellular adhesion and is localised to lateral surfaces of epithelial cells in the region of cell to cell contact that is called as adherens junction. E-Cad plays an important role in establishing and maintaining intercellular connections and by eliciting signals involved in tissue morphogenesis for epithelial integrity and is also involved in various cellular events like polarity, differentiation, growth and cell migration as well as apoptosis. E-cad has the ability to inhibit cell proliferation by up-regulating p27 via epidermal growth factor receptor. Therefore, E-cad is described as major growth or proliferation suppressor biomarker. It is encoded by the gene CDH1 located on chromosome 16q-22 and decreased expression of E-cad has been correlated with cancer progression. E- cad mediated cell-cell adhesion is lost with progression towards malignancy in most cancers of epithelial origin, including head and neck squamous cell carcinoma. 

Though some studies have explored the immunoreactivity of E - cadherin in potentially malignant disorders, non-tumor epithelium adjacent to oral cancer and oral squamous cell carcinoma, it remains unclear if E - cadherin could be used as a bio-marker to predict malignant transformation. We hypothesise the same dilemma and focus only on oral leukoplakia, as it has high malignant potential and thus studied the altered expression of E - cadherin in different levels of dysplasias in oral leukoplakia and the correlation between them.

## Materials and Methods

Ethical clearance was acquired from the Institutional Review board and Institutional Ethical Committee.

*Clinical samples*

Tissue biopsies from fifty leukoplakia patients with varying histological features were collected from patients visiting the outpatient department of SRM Dental College, Ramapuram. A part of the biopsy tissue was stored in formalin to investigate the histopathological features and the other part was saved in RNA save reagent (cat # 01-891, Biological Industries), which helps to keep the messenger RNA intact. The sample tubes were then stored at 4°C for 48h as recommended by the manufacturer, after which the tubes were stored at -20°C until further usage.

*Extraction of mRNA, Quantification and Reverse Transcription (cDNA synthesis)*

Extraction on mRNA was carried out using cat# 740955.50, Machery Nagel, Germany. This kit is capable of extracting even small RNA species such as microRNA and hence was used. 

Quantification of total RNA extracted from the samples was done with QubitTM RNA BR Assay Kit, a fluorescent based RNA quantifying system (cat#Q10210, Invitrogen, USA). 5μl of total RNA was quantified in the presence of 195μl of the quantifying reagent in Qubit fluorescent detection unit (Invitrogen / Molecular Probes, Austria).

Reverse transcription of mRNA was performed using miScript II RT Kit (Cat#218161, Qiagen, Germany) to synthesize complementary DNA (cDNA). This kit was used as it is capable of transcribing both messenger RNA and small RNA molecules with high efficiency and hence was used. 

*Establishment of standards for quantitative real time PCR*

In order to quantitatively determine the copy numbers of E-Cadherin RNA molecules (relative to each other and among the samples), a standard curve was established with serial dilutions of PCR product amplified from human GAPDH (Glyceraldehyde-3-Phosphate Dehydrogenase) gene. Both E-Cadherin and GAPDH amplified with similar efficiency and hence GAPDH was used for generating the standard dilutions. 

After determining the copy numbers, serial dilutions of the GAPDH eluate was made to obtain concentrations from 1 x 10^6^ to 1 x 10^1^. These serial diluted samples were then analyzed by realtime PCR in the presence of QuantiNova SYBR Green PCR Kit (Cat#208052, Qiagen, Germany) in Qiagen 5-plex rotor gene real time PCR system to establish a linear standard graph.

*Real Time Polymerase Chain Reaction*

The primers were designed in such a manner to selectively amplify only the E-Cadherin cDNA but not any contaminating genomic DNA (even when present in smaller amount) during real time PCR reactions. E-Cadherin amplification in the samples was performed using Type-IT HRM Kit (Cat#206542, Qiagen, Germany) in a 20μl reaction. The concentration of E-Cadherin in each sample was expressed as copies per microliter. The copy numbers can be related not only to the presence or absence of E- Cadherin molecules but also to estimate its quantity when present. For example, when a sample contains higher concentration of E-Cadherin mRNA expression, reverse transcription will produce higher amount of E-Cadherin cDNA and vice versa. These samples when analyzed by real time PCR will show a higher copy number of E-Cadherin molecules.

*Statistical Analysis*

Non parametric tests were performed since the data were not normally distributed. The data were expressed as mean and standard deviation (SD). One way analysis of variance with a post hoc Tukey HSD test was used for continuous data. Kruskal Wallis test and Mann Whitney test were used to compare continuous variables between two groups. A two sided p value < 0.05 was considered statistically significant.

## Results

*SqPCR for standards*

To determine the relative copies of E-Cadherin in leukoplakia samples with different grades of dysplastic features, we first established a standard curve by diluting known concentration of GAPDH gene as described in the methods section. Six dilutions of GAPDH samples labelled as s1 to s6 were subjected to real time PCR amplification. Data analysis indicated a linear slope with positive association between the ct (cycle threshold) values and amplifications (copy numbers). For example, an early ct value of 8.39 was observed for a standard with a dilution of 106, while a late ct value of 25 was observed for a standard with a dilution of 10.1.

*qPCR for Test Samples*

Total RNA extracted from samples were first quantified with Qubit fluorometer and 1μg of total RNA from each sample was subjected reverse transcription to synthesize cDNA as described in the methods section. Following cDNA synthesis, primers specific for exonic region of E-Cadherin gene were used to amplify the E-Cadherin molecules present in the samples by real time PCR. Real time analysis showed differential amplification curves for each of the sample among the samples. Comparison of these amplifications with linear standard curve described above resulted in the identification of copy numbers of E-Cadherin present in the samples. Following real time amplification, the samples were subjected to melt curve analysis to determine the specificity of amplification. 

Interpretation of qPCR data: To determine the relative expression of E-Cadherin in samples, the copy numbers of E-Cadherin molecules present in each sample were pooled into four groups: Group 1 (Hyperorthokeratosis / Hyperparakeratosis), Group 2 (Mild dysplasia), Group 3 (Moderate dysplasia) and Group 4 (Severe dysplasia). The copy numbers of samples within each group were then processed to get average of copy numbers. The average copy number values were then used to determine the relative expression pattern E-Cadherin in samples. 

Based on this approach, the expression of E-Cadherin was found to be highest in samples with hyper-ortho or – para keratosis, which was followed by samples with mild and moderate dysplasia. Samples with severe dysplastic feature showed least expression of E-Cadherin. 

Since our study focuses on the expression of E - cadherin in different grades of dysplasia (mild, moderate, severe) we excluded the samples with hyperortho / para keratosis. On statistical analysis, we carried out non parametric tests (Kruskal Wallis) since the mean was lesser than twice the standard deviation.

We reject null hypothesis based on the results obtained from statistical analysis. The result shows statistically significant difference among all the groups at p < 0.001. Mean value of the first group is greater than twice the standard deviation.

**Figure 1:**
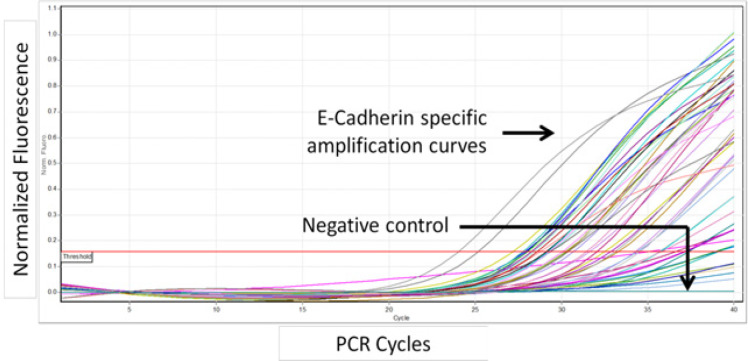
Amplification Curves Obtained from Real Time PCR Analysis to Determine the Concentration of E-Cadherin Expression in Leukoplakia Samples

**Figure 2:**
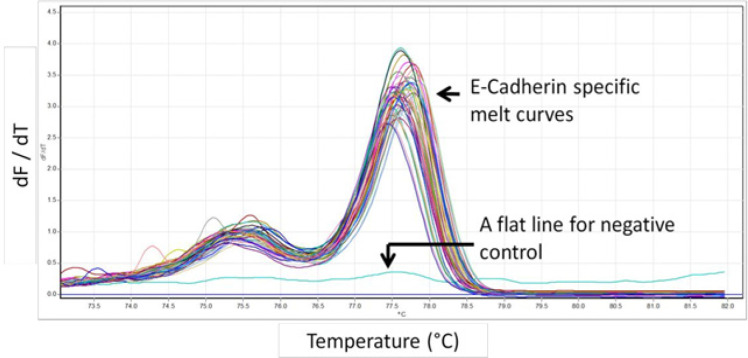
Melt Curve Analysis of E-Cadherin is Shown. E- Cadherin Positive Signals are Displayed as Uniform Melt Curves above a dF/dT Score of 2. Background noise from E- Cadherin negative samples has a baseline dF/dT score of < 0.5. dF/dT indicates rate of change of fluorescence with respect to temperature

**Table 1:** Interpretation of qPCR Data

	Group I (Mild Dysplasia)		Group II (Moderate Dysplasia)		Group III (Severe Dysplasia)		P -Value	Multiple comparisons
	Mean	SD	Mean	SD	Mean	SD		
Calculated concentration	95187.0	17539.084	44448.36	24166.274	15270.67	5551.755	0	I Vs II and III, p< 0.001 II Vs III, p< 0.01
						

## Discussion

Cadherins are cell surface glycoproteins that are responsible for calcium dependent intercellular adhesion during the development of embryo (Halbleib and Nelson, 2006) Cadherins are found in diploblast hydra, choanoflagellates and the sponge Oscarella carmela other than vertebrates, insects and nematodes. Decades after their discovery, it has been proved that E - cadherin also plays an important role in morphogenesis including cell recognition, boundary formation, coordinated cell movements, tissue polarity and also maintains the structure and functions of cells (Halbleib and Nelson, 2006). Hence defective expression of E - cadherin is linked directly with disruption of normal tissue architecture, metastatic cancer, etc. 

Leukoplakias are diagnosed around 4th decade of life and has male prediliction. It is 6 times more common in smokers and is associated with usage of tobacco, alcohol or betel quid. Leukoplakias can be broadly divided into homogenous and non homogenous types. Non homogeneous leukoplakia can further be classified as speckled, nodular and verrucous or exophytic type. Generally leukoplakias are asymptomatic and symptoms when present, are associated with non homogenous type. Proliferative verrucous leukoplakias have higher rate of malignant transformation and it appears as widespread multiple white patches. We can observe different behaviors in leukoplakia based on the habits like betel nut chewing or smoking. The rate of malignant transformation of leukoplakia regardless of the degree of dysplasia varies from 0.13% to 17.9% (Bargale et al., 2012). 

Tissue biopsy was performed as Leukoplakia is a clinical provisional diagnosis. Reasons for performing a biopsy are as follows, (i) to evaluate the grade of dysplasia as it is highly associated with malignant transformation, (ii) to exclude other pathologies including carcinoma which may also present as a white patch, (iii) to exclude other potentially premalignant disorders and (iv) to assess colonisation of candida species within the epithelium (Akhtar et al., 2016) Although there are no contraindications for biopsy, care should be taken for those conditions where biopsy should be done with caution which includes bleeding disorders, uncontrolled diabetes, previously irradiated regions, neurofibromas and any carcinomas as to avoid spread of tumour cells (Kumaraswamy et al., 2012). 

PCR (Polymerase Chain Reaction) was first discovered by Mullis in 1990. It allows detection of pathogens and amplification of specific segment of DNA. It helps in producing multiple copies of DNA or a gene for sequencing, cloning and analysis. We have used quantitative real time PCR in our study to gain information on how much a specific gene or DNA is present in the given sample. We have combined real time PCR with reverse transcription which converts mRNA to cDNA following quantification of cDNA (Garibyan and Avashia, 2013). 

Out of 50 biopsies that was performed 20 were with hyperortho/parakeratosis(n=20), tissues with mild dysplasia were 10 (n=10), moderate dysplasia were 14 (n=14) and severe dysplasia were 6 (n=6). Since our study focused on the expression of E - cadherin with different degrees of dysplasia in leukoplakia, the 20 samples with hyperortho/ parakeratosis were excluded. In the present work, we found that reduced expression of E - cadherin correlates with different degrees of dysplasia in Leukoplakia and also with cancer progression which is statistically proven with a p value of 0.001. Grading of dysplasia is subjective which may have affected the interpretation and results. 

Many authors have studied the expression of E - cadherin in leukoplakia. Kyrodimou et al., (2012) read the expression of E - cadherin and Desmoglein -3 in oral leukoplakia and oral squamous cell carcinoma using immunohistochemistry and concluded that alterations in the expression of E - cadherin and Desmoglein - 3 contributes to the malignant transformation of dysplasia and aggressiveness of the caner. Sridevi et al., (2015) has studied the expression of E - cadherin in oral leukoplakia, oral sub mucous fibrosis, oral lichen planus and squamous cell carcinoma using immunohistochemisty concluding that there are variations in the expression of E - cad but its usage as a prognostic marker is questionable. 

We chose RT - qPCR over immunohistochemistry as computerised methods are being recommended to minimize the problem of subjectivity in visual assessment and scoring of immunohistochemical stained slides (Sinn et al., 2017). More reproducible results with regards to staining intensity are yielded by digital analysis. To our knowledge, this is one of the few studies reported about the expression of E - cadherin in different degrees of dysplasia in oral leukoplakia using quantitative real time PCR which will add on to the evidence in the literature. Though qPCR technique has higher sensitivity and specificity compared to the other conventional methods such as immunohistochemistry, this study was carried out in a relatively small sample size focusing a single premalignant lesion considering the affordability and availability of the PCR kit. 

In conclusion, this study demonstrates the altered expression of E - cadherin in leukoplakia samples with different grades of dysplasia with statistical analyses. Though immunohistochemical technique was used by most of the authors to study the expression of E - cadherin, we have attempted the use of quantitative real time PCR. The molecular characteristics of the gene in different samples were consistent with the results found by other authors. Thus we conclude that loss of E - cadherin can be used as a tumour marker that could determine the susceptibility of normal and potentially malignant tissues to transform into oral cancers. But there are differences in values within the same groups of degree of dysplasia, which may be effected by the duration and frequency of the habit that requires further researches on this aspect. Histological examination revealed hyperortho / para keratosis in few samples which were excluded since it was beyond the scope of our study. To generalise our results, further prospective studies with a large sample size using quantitative real time PCR to read the gene expression should be carried out at multi centre levels.
